# EcoHealth and the Determinants of Health: Perspectives of a Small Subset of Canadian Academics in the EcoHealth Community

**DOI:** 10.3390/ijerph15081688

**Published:** 2018-08-08

**Authors:** Aryn Lisitza, Gregor Wolbring

**Affiliations:** 1 Cumming School of Medicine, University of Calgary, AB 3330 Hospital Drive NW, Calgary, AB T2N 4N1, Canada; 2 Department of Community Health Sciences, Cumming School of Medicine, University of Calgary, AB 3330 Hospital Drive NW, Calgary, AB T2N 4N1, Canada; gwolbrin@ucalgary.ca

**Keywords:** EcoHealth, determinants of health, social determinants of health, health (in)equity, ecological determinants of health, environmental determinants of health, political determinants of health, cultural determinants of health, global health governance, public health

## Abstract

EcoHealth is an emerging field that examines the complex relationships among humans, animals, and the environment, and how these relationships affect the health of each of these domains. The different types of determinants of health greatly influence human health and well-being. Therefore, EcoHealth’s ability to improve human, animal, and environmental health and well-being is, in part, influenced by its ability to acknowledge and integrate the determinants of health. However, our previous research demonstrates that the academic EcoHealth literature had a low, uneven engagement with the determinants of health. Accordingly, to make sense of this gap, our research aim is to better understand the views of a small subset of the Canadian EcoHealth community about EcoHealth and the determinants of health relative to EcoHealth. We used a qualitative research design involving seven semi-structured interviews, which were analyzed using thematic analysis. Our findings suggest a tension across themes and a lack of conceptual engagement with the determinants of health. As we consider a future with rapid, unsustainable changes, we expect the identification and integration of the different types of determinants of health within EcoHealth to be imperative for EcoHealth to attain its goal of improving the health and well-being of humans, animals, and the environment.

## 1. Introduction

### 1.1. Big Picture Problem

We live in a world with varying cultural-political conditions and many social changes including population growth, urbanization, economic growth and development, and technological change [1]. As a result, we see ecological and environmental changes in the form of global and atmospheric change, pollution and ecotoxicity, resource depletion, and loss of habitat, species, and biodiversity [1,2]. The social, ecological, environmental, cultural, and political conditions and changes affect the health and well-being of humans, animals and the environment, reinforcing the idea that health problems are multi-faceted [1,2]. However, efforts to tackle these multi-faceted challenges have been narrowly focused to date [1]. One or few of the social, ecological, environmental cultural, and political conditions and changes are emphasized, often compartmentalizing health issues [2]. EcoHealth is an emerging field [3,4,5] that sees itself as able to overcome this compartmentalization to integrate and address the many determinants that influence the health and well-being of humans, animals, and the environment [2].

### 1.2. The Field of EcoHealth

EcoHealth is an emerging field [3,4,5] and public health effort that strives to overcome the compartmentalization of health issues by drawing researchers and practitioners from different disciplines and emphasizing the importance of integration in addressing health issues [2]. Specifically, the field of EcoHealth aims to “study [the] changes in the biological, physical, social, and economic environments” [6] “to improve the health and well-being of people, animals and the environment” [7]. In pursuing this, EcoHealth relies on transdisciplinarity, participation, and equity, which are the three original methodological pillars as outlined by Lebel in 2004 [8]. The application of such pillars, and the first major use of the term “Ecohealth” was in 2006, at the first International EcoHealth Association’s biennial conference [6].

In 2012, Charron, an influential figure from the International Development Research Centre (IDRC), proposed to expand the three methodological pillars of EcoHealth to six principles: systems thinking, transdisciplinary research, participation, sustainability, gender and social equity, and finally, knowledge-to-action [2]. The first principle known as systems thinking holds that the component parts of a system, in this case humans, animals, and the environment, should not be understood in isolation [6]. Rather, they should be understood within the context of the interactions and linkages among each of the components that make up a system and affect other systems [6]. The second principle is called transdisciplinary research, which demands an inclusive vision of ecosystem-related health issues encountered within EcoHealth and relies on a common framework of blended concepts and theories taken from multiple disciplines and stakeholders such as researchers, community representatives and decision-makers [6]. Participation is the third principle, which refers to the aim of cooperation and collaboration within and across the scientific realm, decision-making groups and the community [6]. The fourth principle known as sustainability, refers to the goal of EcoHealth to protect ecosystems and improve degraded environments to maintain the health and well-being of current and future generations [2]. Social and gender equity is the fifth principle, which seeks to “address unequal and unfair conditions impinging on the health and well-being of women and other disadvantaged groups in society” [2]. The sixth and final principle is knowledge-to-action, which is the idea that the knowledge generated by EcoHealth research must be implemented and applied to improve the environment, and the health and well-being of humans [6].

These six principles are used to examine the complex linkages among society, animals, ecosystems, and (biological and social) environments, in order to understand and improve the health and well-being of humans, animals, and the environment from a local to global scale [2].

### 1.3. The Determinants of Health

Human health and well-being are impacted by numerous factors, many of which are clustered under differing determinants of health such as the social determinants of health (SDH) [9], ecological determinants of health (EDH) [10], environmental determinants of health (EnDH) [11], political determinants of health (PDH) [12], and cultural determinants of health (CDH) [13,14,15]. Within each of these categories, different sets of determinant indicators are outlined to improve health inequity as well as population health and well-being [16,17].

According to the World Health Organization (WHO), the SDH are the “conditions in which people are born, grow, work, live, and age, and the wider set of forces and systems shaping the conditions of daily life” [18]. The Commission on Social Determinants of Health has provided some guidance on the SDH indicators, listing 12 [9]. The Canadian Public Health Association (CPHA) recently generated a list of 65 EDH indicators, which are known as ecological processes and natural resources that are essential for the health and well-being of humans [10]. According to Gnanakan, the EnDH are categorized into (1) the domestic environment including indicators such as water sanitation and food contamination; (2) the local environment, which looks at air, water pollution, and hazardous waste indicators; and (3) global factors such as ozone depletion, climate change and acid rain [11]. The boundary and use of the EDH compared with the EnDH is not clear; there appears to be an overlap in how the CPHA conceptualizes the EDH and how the EnDH are viewed in the literature [1,11]. The Oslo Commission on Global Governance for Health put forward the need to look at PDH, which includes concepts such as norms, policies, and practices that arise from transnational interaction, power asymmetry and global social norms that limit choice and action on health inequity [12]. Finally, the CDH are considered any cultural construct that affects health such as self-determination, individual or collective rights, language and cultural practices, as well as understanding of law and traditional roles [19].

Different types of determinants of health and particularly the interrelationships among them dictate individual and population health [20], therefore efforts to connect the determinants of health are seen as imperative to our ability to address human health issues.

### 1.4. Previous Research

From September to April 2016, we performed a systematized narrative review that aimed to understand the extent to, and ways in which, the academic EcoHealth literature engages with the determinants of health [21]. Our findings suggested that the academic EcoHealth literature does not engage conceptually with “determinants of health” [21]. While the literature did engage with certain indicators of the EDH and EnDH, there was uneven engagement with the SDH indicators and little to no engagement with the PDH and CDH [21]. In addition, there was little conceptual engagement with health (in)equity and global governance [21].

### 1.5. Rationale

Given that the field of EcoHealth aims to improve the health and well-being of humans, animals and the environment [2], and that the different determinants of health and the interrelationships among them largely influence human health and well-being [20], EcoHealth’s ability to improve human, animal and environmental health and well-being is influenced by its ability to identify and integrate the different determinants of health to address human, animal, and environmental health and well-being. Despite this potential, our previous study demonstrated that the academic EcoHealth literature had a low, uneven engagement with the determinants of health, most of which was non-conceptual [21]. To make sense of this gap in the literature, it is of interest to see how academics in the EcoHealth community view the determinants of health and their importance to EcoHealth.

The aim of our study is to better understand the views of a small subset of the Canadian EcoHealth community about EcoHealth and the determinants of health relative to EcoHealth. More specifically, we are examining the ways in which a small subset of academics in the EcoHealth community engage with the determinants of health. To address our research aim, there are three objectives. The first is to describe and characterize EcoHealth academics’ engagement with the determinants of health. The second objective is to examine the ways in which academics describe the determinants of health relative to EcoHealth, and the third is to provide recommendations on a fuller uptake of the determinants of health in EcoHealth.

## 2. Methods

### 2.1. Analytic Framework

**Figure 1:**
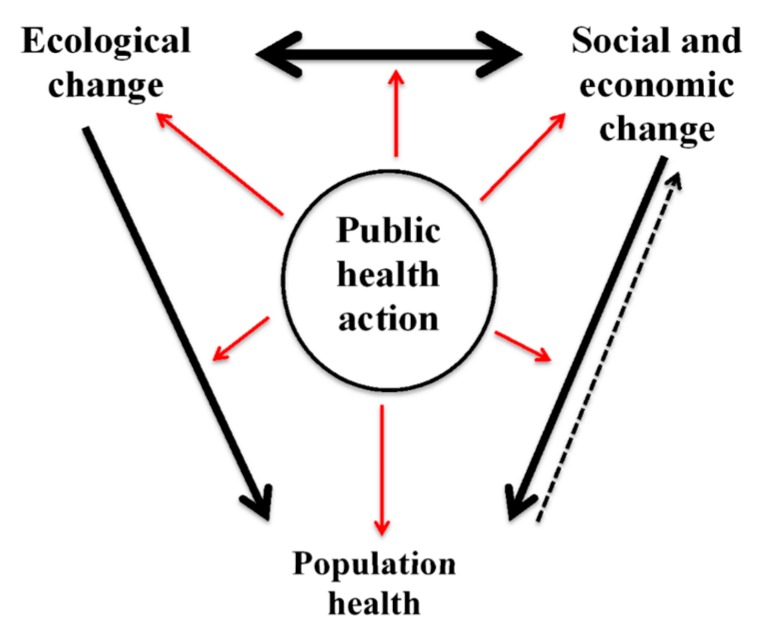
An Eco-Social Framework for Public Health Action (reproduced with permission from the CPHA) [10].

We chose the CPHA’s eco-social framework because it and the CPHA Policy Brief emphasized (1) the social and ecological changes, and EcoHealth as a possible foundation from which to connect the two; (2) the role of public health in addressing these changes; and (3) the implications for population health and well-being (Figure 1) [10].

### 2.2. Research Design

Our research is informed by a qualitative paradigm [22]. Our ontological assumptions about what can be known are grounded in critical realism, while our epistemological assumptions on how we come to know things lie in post-positivism [22]. Our ontological and epistemological assumptions led us to a qualitative research design [23] to explore how academics who are a part of the EcoHealth community view and engage with the determinants of health. A qualitative research design is most appropriate for our project because we would like a more in-depth understanding of academics’ community-specific views and experiences [22].

We undertook an experiential qualitative approach, accepting and prioritizing the views, perspectives, experiences and practices of those interviewed in the Canadian EcoHealth community [22]. Our approach was inductive, data-driven, and bottom-up, linking themes to the data [22].

To collect the data, our method was virtual semi-structured interviews [22]. We chose interviews as our method of data collection because they are best suited for exploring understandings and perceptions that participants have a personal stake in [22], in this case in EcoHealth. Semi-structured interviews also allowed us to guide the conversation towards our research question, and yet permit participants the opportunity to discuss issues that are important to them [22].

To conduct research with humans, we must consider the four ethical principles of justice, autonomy, maleficence and beneficence [24]. As follows, the study was approved by the Conjoint Health Research Ethics Board at the University of Calgary on 11 August 2016—Ethics ID: REB16-1124.

### 2.3. Participants and Sampling

The participants were recruited using expert snowball sampling, a form of purposive sampling [23]. We reached out to one EcoHealth academic by email, followed by another three in-person at a research conference in Calgary, Alberta. We had these four EcoHealth academics reach out to other colleagues and wider EcoHealth networks across Canada to participate in our project.

We chose expert, snowball sampling because it involves selecting participants on the basis that they will provide ‘information rich’ accounts [22]. Our inclusion criteria consisted of those who are members of the EcoHealth community in Canada. We targeted this relatively homogeneous group of Canadian EcoHealth community members because we believe that pre-existing EcoHealth knowledge and experience is necessary to understand and comment on the importance of determinants of health in relation to EcoHealth.

### 2.4. Data Collection and Storage

We developed an interview guide to elicit information regarding how those within the Canadian EcoHealth community view EcoHealth and the determinants of health relative to EcoHealth (Appendix A). The interview guide consisted of open-ended questions and probes on the following broad sections: (a) demographics; (b) EcoHealth; and (c) the different determinants of health and their individual indicators. The majority of the interview questions were descriptive, evaluative or direct [22] (Appendix A).

Informed and voluntary verbal consent was obtained from each participant at the beginning of the interviews. Six semi-structured interviews were performed via Skype and one via phone, each of which ranged from approximately 48 to 75 min long. Each interview was recorded using a digital audio recorder to transcribe and later analyze the data. Interview recordings, transcripts and notes were securely stored in the Health Sciences Centre of the University of Calgary.

### 2.5. Data Analysis

Using Express Scribe^®^ playback software, each interview recording was orthographically transcribed into Microsoft^®^ Word; an audio-style of transcription that focuses on what was said rather than how it was said [22]. To protect the privacy and confidentiality of the participants, each of them was coded for by “Participant 1, Participant 2 etc.”, and all identifiable information was anonymized. The interview transcriptions were then uploaded in a PDF format into ATLASti-7^®^, a qualitative data analysis software, where we conducted a thematic analysis [25].

#### Thematic Analysis

We performed a thematic analysis [22,26] for this project because we wanted (1) an inductive and iterative approach conducive to identifying themes in a ‘bottom-up’ way; (2) an approach that permitted us to understand, in-depth the patterns or themes in the responses; and (3) flexibility in terms of a theoretical framework in order to discuss relevant theories or research findings after analysis [22]. Accordingly, we employed the following systematic, six-phase thematic analysis process described by Braun and Clarke [26].

Phase One is data familiarization whereby the researcher re-reads the transcripts to learn the content as well as to start identifying interesting features [26]. The generation of initial codes is Phase Two, which entails identifying meaningful bits of data [26]. We used semantic coding, which “…[is] more grounded in the data, and prioriti[zes] the meanings provided in the data” [26]. Phase Three, identifying themes, involves determining broader patterns of meaning in relation to the research question [22,26]. In Phase Four, called reviewing the themes, the identified codes and themes are revised to ensure that the themes represent the coded data, that they are meaningfully distinct and that there were no missing patterns [26]. Defining themes is Phase Five whereby the essence of each theme is identified [26]. The last stage, producing the report, involves the final analysis and the write-up of the report [26].

### 2.6. Trustworthiness Measures

To enhance credibility, we engaged in members checking whereby the interviewer rephrases what the participant is saying during the interview to check that their voice is being represented accurately [27]. To enhance dependability [28], we generated audit trails within ATLASti-7^®^ to describe the decisions and reasoning that guided our research.

## 3. Results and Discussion

Our participants were mostly females whose ages ranged from late twenties to late forties (Table 1). At the time of data collection, each participant had taken up residence in Canada, with a small majority residing in Western Canada (Table 1). The educational background of the participants was varied and included formal education from the disciplines of science, human ecology, medical education, sociology, political science, health sciences, environmental studies, recreational therapy, nursing, community health and most commonly public health (Table 1). Looking at occupation, participants were graduate students, researchers, or postdoctoral fellows or professors, all of which were part of the wider academic EcoHealth community (Table 1).

In what follows, we identify five themes from the Canadian EcoHealth community members’ views on EcoHealth and the determinants of health (Table 2). In line with our first and second research objectives, we describe how EcoHealth academics engage with the determinants of health and the determinants of health relative to EcoHealth. We then discuss the remaining cross-cutting themes, the connections across them and the implications of these connections according to our previous literature review results. This is followed by the application of the CPHA framework and vision to address our third research objective.

Data extracts from the participants’ transcripts are included to illustrate some of the views expressed in relation to specific themes. Data extract disfluencies were removed to improve readability.

### 3.1. The Determinants of Health and the Determinants of Health Relative to EcoHealth—Objectives 1 & 2

Theme 1: Recognition of Definitional Issues—The Determinants of Health

Participants defined the determinants of health as factors that influence or determine one’s health, as well as the capacity to attain one’s goals. In a few cases, the determinants of health were critiqued as linear pathways rather than systematic ones, and as more downstream rather than upstream influences. Participants’ accounts of the determinants of health extended beyond the literature, which casts the determinants of health as factors that influence our health, or the ‘causes of the causes’ [29]. 

Subtheme 1.1: The Social Determinants of Health

Participants were most familiar with the SDH. The SDH were seen as a form of attention to equity and health, aspects of belonging, a representation of class in society, and most commonly, daily living conditions or life circumstances, which is in line with the WHO [18]. Participant 2 comments:

P2: …in the 2008 report of the Commission of the Social Determinants of Health, they talk about the daily living conditions…

The SDH were also defined as actors that help the EcoHealth community to work well within a complex system, social and structural pathways of influence, and things that are outside of the body that affect health. The most common individual SDH indicators that were mentioned were housing and income or income inequality.

Participants stated that EcoHealth engages with the SDH by assuring that scholarly work is informed by the SDH, engaging with the SDH indicators at the project level, and focusing on the SDH through the principles of social and gender equity, sustainability, and systems thinking. All participants discussed the use of the SDH and EDH together, and their specific indicators within EcoHealth and their own EcoHealth research projects, which is well-aligned with the CPHA’s eco-social framework [1]. Participant 7 indicates this:

P7: I’m very regularly describing my research, which I would consider as EcoHealth research, to be addressing the social and environmental determinants of health together.

Other participants stated that EcoHealth should engage with the SDH by engaging not only with the social and ecological facets, but with the wider political, spiritual, physical and cultural interplay as well as the forces upstream from the SDH respectively. This view blurs the distinction between the different determinants of health. Participants believed that EcoHealth engaged most with the SDH indicators of food and nutrition, Indigenous and health services. However, several participants said that the SDH indicators that EcoHealth engages with will depend on the specific research project and that EcoHealth should engage with each of the SDH indicators.

Subtheme 1.2: The Ecological and Environmental Determinants of Health

Sub-subtheme 1.2.1: The Ecological Determinants of Health

The EDH were commonly defined as systematic, broad and interrelationship-focused. They were said to reflect complex systems including nonhuman organisms, ecosystem factors that determine your health, global issues and processes, as well as how human societies or systems impact the environment or health systems. This thought is captured by Participant 6:

P6: …I would assume ecological determinants of health to be more how human societies are interacting with the environment, and the results and consequences there…

Of the EDH indicators, climate change and bio-physical changes or factors were mentioned the most.

According to participants, EcoHealth engages with the ecological determinants through an eco-systemic approach, in focusing on upstream factors to health, the notion of stewardship, and the belief that other entities have intrinsic value. Participants also stated that EcoHealth should engage with the EDH in a way that is consistent with systemic ecosystem approaches to health and to engage in reciprocal maintenance with the inclusion of other species, as shown below:

P7: …in its most integrative framing, I think [the] ecological determinants of health can embrace multiple relationships and the notion of um reciprocal maintenance…

Participants identified climate change, agricultural practices and watershed management as the individual indicators that EcoHealth engages with, while other participants stated that the indicators with which EcoHealth engages with would depend on the research project.

Sub-subtheme 1.2.2: The Environmental Determinants of Health

The EnDH on the other hand were described in the least variable way of each of the determinant types. All participants defined the EnDH as biophysical pathways of hazards or contaminants. Parallel to what is found in the literature, Participant 7 states that without this language, it would be difficult to conceptualize the EnDH [1,11]:

P7: I think the environmental determinants of health are a little bit poorly conceived. I would struggle, other than using those languages of hazards and contaminants, to list the environmental determinants of health.

Participants stated that the way in which EcoHealth engages with the EnDH would depend on the individual researcher or practitioner. However, several participants identified EcoHealth’s engagement with the EnDH as non-systemic, which is demonstrated below:

P2: Some studies are quite environmental in the sense that they don’t really involve a lot of examining ecosystem dynamics…

The individual EnDH indicators were commonly listed as water and soil. Participants mentioned that the individual EnDH indicators that EcoHealth engages with will be dependent on the research project, although specifically, EcoHealth engages with water as an individual indicator.

Sub-subtheme 1.2.3: The Ecological/Environmental Determinants of Health

In three cases, the participants viewed both the EDH and EnDH as being the same. These determinants were defined broadly as the physical, cultural, political, social and spiritual constructs, or the things in the environment that influence health, as demonstrated below:

P3: … [they are] the things in the environment that are influencing our health, such as the air we breathe…

Evidently, the boundaries between the different determinants are unclear. Participants also identified the EDH/EnDH indicators as many different factors including social processes, pathogenic organisms and ecosystem functioning.

EcoHealth was said to engage particularly with water, sustainability and land, while several participants said that EcoHealth should engage with all the EDH/EnDH. One participant said that EcoHealth engages with the EDH/EnDH by addressing ecological change in the context of social and economic change, which invites integrative thinking and transdisciplinarity, while another thought that EcoHealth work gave primacy to the physical and that this was problematic:

P5: In terms of our study, do we give primacy to physical examinations of water? Yea we do. Is that a problem? Yes, it is.

Each of the three participants who defined the EDH and EnDH as the same, believed that EcoHealth should engage with these determinants in an eco-systemic way.

Subtheme 1.3: The Political Determinants of Health

The PDH were most commonly defined as governance, particularly governance structures and forms of decision-making. The PDH were also defined as contextual political systems, power, policy, and interest groups. Participant 2 touches on a number of these factors, linking the PDH to the literature [12]:

P2: There’s political determinants of health in the Lancet commission. As far as I can tell, it’s probably factors that involve policy and political decisions and the interest groups that affect them…

Notably, several participants believed that the PDH are closely related to or part of the SDH, as mentioned by Participant 7:

P7: I see political determinants of health in some ways as very closely related to the structural social determinants of health.

Participants identified the individual PDH indicators as policy, political system, power structures or dynamics, legislation and government.

According to participants, EcoHealth engages with the PDH through the consideration of power relationships, legislation, political actors, social justice, and policy recommendations. However, participant 7 found EcoHealth to be politically naïve:

P7: …some people would argue that EcoHealth work can be extremely politically naïve, particularly in relations of dynamics of power and equity.

Different participants believed that EcoHealth should not be politically naïve, and that EcoHealth should engage with upstream factors and advocacy. One participant said that EcoHealth must not engage with the individual PDH indicators in isolation, while other participants said that EcoHealth should engage with the specific PDH indicators of ideology and colonial legacy. 

Subtheme 1.4: The Cultural Determinants of Health

The CDH were defined as the ways in which culture, including your practices, behavior and beliefs, influence health. Participant 6 speaks to cultural practices:

P6: I’m thinking that it’s things people do that are cultural practices that would specifically impact their health…

Participants also identified the CDH as being associated with ethnic groups. However, within these ethnic groups, one participant stated that it is upstream factors rather than culture that causes illness:

P2: …[people] say it’s their culture that’s making them sick [but] that’s a way to ignore the upstream factors that are creating problems for that group…

The most common individual CDH indicators were outlined by participants as cultural beliefs or practices, intergenerational knowledge and the experiences of culture, food, gender, and childhood.

Participants said that EcoHealth engages with the CDH through the acknowledgement of different knowledges, equity dynamics, participation, and transdisciplinarity. According to participants, EcoHealth should be engaging with the CDH by working with many different cultures, looking at upstream factors, practicing multi-culturalism acceptance and acknowledging the cross-culture value of the environment. Participants stated that EcoHealth engages with the specific indicators of spiritual health and cultural practices. Again, one participant mentioned that the indicators EcoHealth engages with are context-dependent.

Subtheme 1.5: Recognition of Definitional Issues—A Synopsis

Participants were most familiar with the SDH as social pathways of influence [18]. Participants were also familiar with the EDH and EnDH and most participants articulated a difference between the EDH and EnDH. The EDH were understood, similar to the CPHA definition [1], as systemic understandings and interrelations, while the EnDH were seen as contaminants and hazards, in line with Gnanakan’s EnDH [11]. The EnDH were discussed as being part of EcoHealth work, but certainly not a priority given their non-systematic and often narrow focus. The dichotomy however, between the EDH and EnDH, was not represented in participants’ discussions of the individual indicators. There was significant overlap between the EDH and EnDH determinant indicators, which is in line with the CPHA [1]. Several participants believed that the PDH and CDH were a part of the SDH and should not be considered as separate conceptually, which is contrary to the Oslo Commission on Global Health Governance and the Lancet [12]. Participants highlighted the importance of engaging with the PDH in EcoHealth work, although they could not always articulate in what ways and which indicators may be involved. One simply indicated that EcoHealth was blind to the PDH, which relates to the ‘Blind to Political Power’ subtheme. Few participants could conceptually discuss EcoHealth’s engagement with the CDH or CDH indicators, and if so, participants often gravitated to minority groups or listed several WHO SDH indicators [9]. All participants clearly emphasized the importance of engaging with each type of determinant within EcoHealth, even if there was a lot of ambiguity and overlap across the determinants. All seven participants also saw little value in ranking the different types of determinants of health and strongly emphasized the interconnections among the determinants, which is in line with the theme of ‘The System’.

### 3.2. Connections Across Themes Arranged According to Previous Research—Objective 2

#### 3.2.1. Uneven Engagement with the Determinants of Health

A previous finding noted that, while there was coverage of the SDH, the coverage across the individual indicators was uneven, and there was little to no engagement with the PDH and CDH [21]. This finding was reproduced in our current study. Participants expressed that majority of their research is driven by local community health concerns or complex health issues. While this community-based focus is valuable, it suggests that what is researched, and the determinants of health that are engaged with, is dependent on the dominant research interests and prevailing funding opportunities. This pattern leads to a more uneven and less predictable engagement with the determinants of health that is dependent on which projects are seen as useful at any given time. Given this pattern, it is understandable that certain determinants of health types and indicators such as the PDH and CDH are less visible, while others including certain SDH indicators and forms of the EDH and EnDH are more prominent. However, this form of engagement with the determinants of health similarly does not acknowledge each of the different types of determinants of health and the interrelationships among them; failing to realize, and build on, the important role that the determinants of health play in helping EcoHealth to attain its goals.

Theme 2: The System

A predominant concept that transcended each interview is the idea of a set of connected parts that form a complex whole known as a system.

Subtheme 2.1: Ecosystem

Ecosystems came up frequently in discussions of ecology, and they were tied explicitly to EcoHealth through the phrase ‘ecosystem approaches to health’. The term ecosystem was used frequently to speak to the difference between ecology and the environment, a comparison that is key in the distinction between the EDH and EnDH. Participant 7 situates this discussion:

P7: …environmental determinants health [are] often focused on hazards and risk, and the ecological determinants of health are often focused on an understanding of ecology and ecosystems as a home. That difference between hazard and home has some profound implications for me in terms of how we do our work.

This distinction is debated [1,11]. Irrespective of whether the two are distinct conceptually, it would be beneficial for the field to use consistent language to establish a more concrete way of understanding and addressing these determinants.

Subtheme 2.2: Integrating Individual Elements 

EcoHealth was seen as a way to connect, relate or integrate elements of a system. Participant 1 states:

P1: …it’s that kind of integrative element, drawing connections, that in my mind is really essential when we’re thinking about EcoHealth…

When asked about an achievement of EcoHealth, several participants responded that it is EcoHealth’s ability to integrate elements of a system. Participants also described the importance of considering each of the determinants of health within a system. In doing so, most participants were uncomfortable with separating or prioritizing the different types of determinants of health. While addressing the individual indicators for the PDH, Participant 2 highlighted:

P2: …when you list them as individual things, you’re obscuring their possible interconnections, and separating them seems like kind of an arbitrary or artificial thing that won’t necessarily be productive.

The consistent reluctance of participants to acknowledge and prioritize specific determinants of health indicators ties into the idea of ‘systems thinking’. 

Sub-subtheme 2.2.1: Systems Thinking and Understanding

Eco-systemic or systems thinking was seen as an orientation to the research that is specific to EcoHealth. Systems thinking holds that the component parts of a system, in this case, humans, animals, and the environment, should not be understood in isolation [6]. Rather they should be understood within the context of the interactions and connections among each of the components that make up a system [6]. Systems thinking was viewed as important in the meaning, purpose, and future of EcoHealth, and one participant said that EcoHealth should continue to seek an eco-systemic understanding of what is going on. Participant 1 discusses the use of systems thinking in EcoHealth:

P1: In true system thinking fashion, it’s about the relationships between those components parts rather than just thinking about one particular outcome or one particular driver of change.

Systems thinking was described by participants as the actual or intended approach to the concept of the determinants of health, the different types of determinants and the determinant indicators. As mentioned above, participants often took a systems approach, preferring not to view the different determinants in isolation from each other.

Theme 3: Multiple Realities

Many participants reflected the assumption of multiple realities within their responses concerning EcoHealth and the determinants, which comes from a relativist ontology. Relativism dictates that there are multiple realities rather than one single truth, and that we may not go beyond these realities [30]. The epistemological assumptions that follow a relativist ontology are grounded in social constructionism. Social constructionism argues that the world and what we know of it do not reflect one truth, but rather, knowledge is constructed through various situated systems of meaning [22]. Therefore, participants’ responses were often fluid or ambiguous.

Subtheme 3.1: ‘It Depends’

All seven participants talked about how the answer to certain questions would depend on the research question, the researcher, and the context of a project. Similarly, this view recognizes that each EcoHealth project has a different way of thinking, doing, and relating to the determinants, which will construct knowledge differently than other projects. Participant 1 states:

P1: …the degree to which that’s done well in EcoHealth communities again depends on the project, depends on the people who are running it, depends on the kinds of questions that they’re asking.

Similarly:

P6: I find that [EcoHealth engagement] varies considerably according to the scholar and also the particular research project that they’re working on.

Consequently, the discussion around the definition of EcoHealth and the individual determinant indicators was often said to be researcher-dependent. This is one of many factors that contribute to a disjointed and uneven engagement with the determinants relative to EcoHealth.

Subtheme 3.2: Normative

The word normative is used to describe something that is established, related or derived from a standard or a norm. The term normative implies that one’s perspective may be different depending on the norms that were constructed in a particular context and thus, there is more than one reality. Several participants acknowledged the normative nature of certain determinant questions or issues, while one participant provided a more direct account of normativity:

P1: …the question that you’re asking is one that is inherently normative and for me it’s a no brainer but for others, they might say no, other things are more important…

The ‘Normative’ subtheme is similar to the ‘It Depends’ subtheme in that it can contribute to an uneven engagement of the determinants relative to EcoHealth.

Subtheme 3.3: Labelling

Several participants expressed a discomfort with labelling or branding EcoHealth as a distinct, discrete or boundable entity with rigid boundaries and expectations. Rather, participants saw EcoHealth as an integrative space that explores the determinant interconnections among humans, animals, and the environment. Participant 7 addresses this conflict while talking about their vision for the future of EcoHealth:

P7: Yea, the question around definition is deeply challenging for the field…

A: Mhm.

P7: …there are multiple definitions for EcoHealth…

Participants’ fluid view of EcoHealth conflicts with the IDRC’s efforts to outline EcoHealth as its own definable field with distinguishing goals and six specific guiding principles from which to achieve those goals [2]. Further, participants were again not interested in acknowledging the different types of determinants as their own entities. I highlight this in a quote by Participant 2 on the PDH:

P2: …this was a question that when I read it, I felt a little uncomfortable because I don’t know that there are ‘the’ political determinants of health.

This viewpoint contributed to participants’ difficulty to conceptually define the different determinants.

#### 3.2.2. Conceptual Engagement with the Determinants of Health

Looking at our previous study on the extent, and ways in which, the academic EcoHealth literature engages with the determinants of health, our findings had suggested that the academic EcoHealth literature does not engage conceptually with the determinants of health [21]. As noted above, this previous finding is paralleled in participant accounts. These results can be explained by participants’ construction of ‘Multiple Realities’ as a theme and their strong focus on EcoHealth in practice. Participants highlighted that they did not want to label the field or related concepts as specified units for grouping and understanding phenomena. This reluctance to label is part of a larger anthropological academic movement that seeks to rebuild our understanding of environments on constructionist ontological foundations [31,32,33]. The purpose of the movement is to better acknowledge the totality of relations existing among people and their environments, and to better respond to our system as a whole [31,32,33]. Participants also viewed EcoHealth as a community or field of practice and did not value conceptual and theoretical development as compared to practical applications. This practical prioritization is well-aligned with EcoHealth’s strong focus on putting the EcoHealth principles into practice and the specific IDRC EcoHealth principle of knowledge-to-action, which focuses on translating research into applications and action [2]. The result of this academic drift away from labelling and strong focus on community practice is that the field itself and many of the different types of determinants of health were not discussed in a concrete or conceptual way by participants. The consequence of these conceptual gaps in the coverage of the determinants of health is that the EcoHealth community is not able to identify and integrate each of the determinants that affect the health of humans, animals, and the environment; acting as a barrier to EcoHealth’s goal of understanding and improving interactions at the human-animal-environment to health interface. Participant 2 adds that there is a lack of understanding, enthusiasm and funding for EcoHealth, which could be in part, a result of these conceptual limitations. 

Theme 4: Power

Power invokes a capacity or ability to direct or influence others or the course of events. One participant brought up the importance of power in the research process of EcoHealth and the determinants of health work, which is exemplified below:

P1: …taking a good, hard look at the kinds of research processes that you’re using: who’s being engaged, how early, how much power do they have over defining the research question, research processes, how much ownership do they have over the data…

Power is exhibited differently by different stakeholders in EcoHealth.

Subtheme 4.1: Blind to Political Power

One participant stated that EcoHealth was dangerously blind to the PDH, which according to participants, includes power:

P7: …being blind to the political determinants of health is very dangerous for EcoHealth work and it’s probably considered to be a critique…

A second described EcoHealth as politically naïve, while a third stated that EcoHealth does not, but should, look upstream to large scale political forces:

P2: …my vision will be that [EcoHealth] pay[s] more attention to upstream political and economic power…

When there exists ‘multiple realities’ and viewpoints in one field, it is important that the community acknowledges and uses their governing and political forces towards common goals.

Subtheme 4.2: Colonialism

The Canadian history of colonialism and the extension of power and control over other groups plays a role in health research. Participant 7 identifies this:

P7: …in Canada, we rub up against the political determinants of health in the context of colonial process and history, and long-standing intergenerational dynamics about where power and control lie and how those things influence [health and health research].

Those most affected by colonialism in Canada are the First Nation’s, one of many groups encompassed in the SDH [18]. One participant noted a different post-colonialist power structure in First Nation’s communities, while another noted the ongoing effects of colonialism, which is shown below:

P3: …looking at First Nations rights and industry, the politicians ultimately have the power to decide which industrial projects go ahead…

#### 3.2.3. The Issues of Health (In)equity and Global Health Governance

A final finding from our previous work focused on the lack of engagement with key concepts such as health (in)equity and global governance of health [21]. Our results from this study also suggest that there was little to no engagement with health (in)equity [21]. Only one participant mentioned health (in)equity, regarding the implications of natural gas development and climate change. This is surprising given that health (in)equity is key to the determinants of health and to EcoHealth as part of the public health sphere [2]. Additionally, our results similarly suggest a lack of engagement with global governance of health, also captured by the term ‘global health governance’ [21]. This issue is recognized in the ‘Power’ theme, whereby Participant 7 outlines the need for the EcoHealth community to better understand political processes and governance as in who is included in, or excluded from, decision-making and where the power flows. Furthermore, one participant expands on this thought to argue that EcoHealth work can be extremely politically naïve, particularly in relations of dynamics of power and equity. The Oslo Commission on Global Governance for Health argues that global governance activities involving different actors and forms of power fails to protect public health and can often be the cause of health inequities [12]. An example of this is the ‘Colonialism’ subtheme, which demonstrates that power imbalances between different groups affects health and creates health inequities. As participants highlighted, the colonial process and its history still exists in Canada and it continues to contribute to health inequities. Again, participants’ focus on local community-based health issues and reluctance to conceptually label or ground EcoHealth and its goals, resulted in poor coverage of key areas such as health inequity and global health governance; leaving no conceptual guide from which to influence the EcoHealth community and wider sphere of stakeholders and political actors. The implication of this is that the EcoHealth community is likely not fully addressing these issues, which are necessary to achieve the goals that EcoHealth seeks to attain.

Theme 5: Transdisciplinarity

Transdisciplinarity demands an inclusive vision of the complex issues encountered within EcoHealth and relies on a common framework of blended concepts and theories taken from multiple disciplines and stakeholders such as researchers, community representatives, and decision-makers [6]. Five of seven participants discussed transdisciplinarity in-depth. 

Subtheme 5.1: EcoHealth and Transdisciplinarity

EcoHealth itself was seen as transdisciplinary. Participant 2 mentioned:

P2: EcoHealth is transdisciplinary, so there will be people who try to overcome those disciplinary boundaries…

Transdisciplinarity across disciplines and ways of knowing was discussed as both an achievement and a necessary component for the future of EcoHealth. Participant 4 stated:

P4: …I think a benefit or sort of a more unique aspect of [EcoHealth] is the opportunity for interdisciplinarity and transdisciplinarity in doing EcoHealth, and having people working across different projects that have different topics…

Indeed, EcoHealth is a diverse community. However, as mentioned above, there is a lack of common terminology regarding EcoHealth or other important concepts within EcoHealth for different stakeholders to successfully employ.

Sub-Subtheme 5.1.1: Public Health

One participant noted EcoHealth’s transdisciplinary pairing and engagement with public health. Participant 3 stated:

P3: I think from my perspective, a big opportunity for EcoHealth has been it’s pairing with public health, and how people are starting to realize that EcoHealth can really help the public health field, it’s not in competition with it.

In the literature, EcoHealth is often viewed as part of the public health sphere [1,2]. 

Subtheme 5.2: Different Knowledges

EcoHealth’s engagement with different knowledges was viewed as transdisciplinary. Similarly, one participant described bringing different knowledges together to address complex problems in a transdisciplinary way:

P7: Thinking about transdisciplinarity is a way, not just to bring disciplines together, but to bring different knowledges together to respond to a complex problem, [which] is really useful…

Another participant addressed the implications of a transdisciplinary community or field of practice such as EcoHealth. Participant 1 noted:

P1: …it’s a struggle or a challenge for a transdisciplinary community in practice or [a] transdisciplinary field of practice in so far as you’re going to have people who have a fundamentally different orientation towards science, and so there’s a whole slew of challenges that are associated with that…

As was mentioned above and is reflected in the ‘Multiple Realities’ theme, participants’ differing orientation to science presents challenges in terms of establishing and utilizing well-understood, blended concepts within the EcoHealth field. 

Sub-Subtheme 5.2.1: Traditional Ecological Knowledge

Traditional ecological or Indigenous knowledge refers to the long-term and evolving knowledge that local or Indigenous groups hold through their direct relationship with the environment. One participant describes EcoHealth as a way to articulate this traditional ecological knowledge or what they term as ‘ancient wisdom’. Another participant articulates EcoHealth’s consideration of traditional Indigenous knowledge:

P3: EcoHealth has worked with looking at the value of Indigenous knowledges and returning to traditional ways...

Traditional ecological knowledge is one of many forms of knowledge that is valued by the EcoHealth community.

#### 3.2.4. A Key Tension

In both the IDRC conception of EcoHealth and alternate conceptions, the EcoHealth community sees itself as a place for inclusion and integration [2]. Participants reinforce this idea in their discussions of the importance of integration across component parts of a system and across disciplines or ways of knowing as is reflected in the themes and IDRC principles of ‘The System’ and ‘Transdisciplinarity’ respectively [2]. Participants also demonstrate this indirectly through the theme of ‘Multiple Realities’, which outlines a reluctance to distinguish, label or prioritize certain concepts or entities to leave space for the inclusion of concepts or entities other than those that are labelled. Despite this focus, our previous and current results indicate large gaps in the coverage of the different types of determinants of health and their indicators [21]. There is a curious, underlying tension between efforts to hold a platform for integration and the exclusion of important or common concepts and understandings. In other words, the EcoHealth community’s attempt for inclusion and integration, and the resulting opposition to conceptual labelling and structuring, allows the EcoHealth community and its wider political forces to take a more narrow, unspecified focus, fostering low and uneven engagement with concepts that are important to EcoHealth and its goals. In this case, there is poor engagement with many of the determinants of health and the interconnections among them [21]. This engagement is concerning given that EcoHealth’s ability to achieve its transdisciplinary mandate to improve human, animal and environmental health and well-being is influenced by its ability to identify and integrate the different determinants of health across multiple stakeholders. Therefore, while we must continue to prioritize integration and a systemic orientation, it would be beneficial to put forward a framework to ground and guide the EcoHealth community on what determinants of health to consider while conducting EcoHealth research. The framework is intended to be acknowledged and used where possible or appropriate rather than rigidly adhered to; we would like to maintain a space for new or alternate determinants, concepts and transdisciplinary forms of action.

### 3.3. Application of the Canadian Public Health Association Policy Brief—Objective 3

According to the CPHA’s Policy Brief, we live in a socio-ecological system with a human-created social system and the natural environment [10]. The social system undergoes frequent changes which shape the conditions of daily life and is therefore recognized as part of the SDH [10]. The natural environment provides ecosystem services that the CPHA calls the EDH, generating 65 indicators, which are fundamental to health [10]. Accordingly, the CPHA developed a model that emphasizes both the “ecological and socio-economic determinants, their interactions, the implications of change for the health of the human population, and the role of public health in responding to these circumstances” (Figure 1) [10].

However, certain social changes are harming the ecological determinants, and posing a global threat to human health [10]. The CPHA’s Policy Brief states that in order to address this global threat we must reconnect the SDH and EDH, claiming that EcoHealth is one foundation in which this reconnection can occur [10]. Indeed, EcoHealth sees itself as able to overcome siloing to address the many determinants that influence health and well-being of humans, animals, and environment [2].

**Figure 2:**
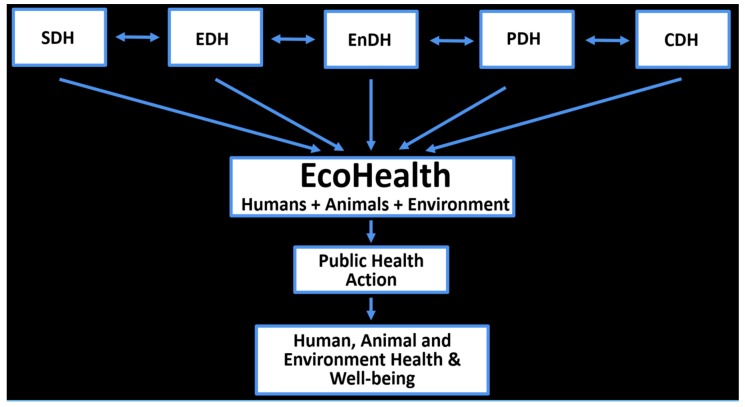
New Analytic Framework on the Determinants of Health and EcoHealth.

Given EcoHealth’s potential to identify and integrate the different determinants of health to improve human, animal, and environmental health as well as our findings on the ways in which participants engage with the determinants of health, we propose the following model (Figure 2).

To begin, we suggest that it would be beneficial to explicitly emphasize the different determinants of health and their specific indicators, including the SDH, EDH, EnDH, PDH, and CDH (Figure 2). Indeed, the creation of the CPHA Policy Brief was driven from the realization that there was a need to highlight global environmental changes, many of which were reflected in what they outlined as the EDH, because there were many things that were not sufficiently covered under the indicator of ‘physical environment’ as part of the SDH [18]. Similarly, we see it as important to go beyond the SDH, EDH and EnDH to explicitly list the PDH and the CDH, which according to the Oslo Commission and the Lancet, are important facets that determine health and health equity [12,19]. The explicit mention of the PDH and CDH are also needed to fulfill EcoHealth’s transdisciplinary mandate. 

Secondly, we propose that EcoHealth becomes a key player with each of the different types of determinants of health including the SDH, EnDH, EDH, PDH and CDH, integrated within and informing EcoHealth (Figure 2). With EcoHealth’s focus on humans, animals, and the environment, it can expand the human-centric concept of the determinants of health beyond the human domain. The exploration of these different types of determinants across humans, animals, and the environment within EcoHealth would inevitably affect public health policy and action, which would directly affect the downstream health of these three domains (Figure 2). There are many other forms of cross-sectoral action and feedback that could be affected or have an effect, across several different policy domains, however here we simply layout the key set of interactions from which to build on.

In line with the original CPHA eco-social framework (Figure 1), academics in the EcoHealth community engaged with the connections among the SDH and EDH. In some cases, participants referred to the CPHA’s Policy Brief and in other cases, participants directly stated that EcoHealth research addresses the SDH and EDH together. One participant believed that the ecological and social facets of a system are so fundamentally intertwined that the language which separates them represents a false dichotomy. However, participants did not represent a vision beyond this to include not only the SDH and EDH, but also the integration of the EnDH, PDH and CDH within EcoHealth as reflected in our modified version of the CPHA model (Figure 2). Participants could also elaborate on the actual or perceived distinction between the EDH and EnDH. Given the lack of conceptual engagement, the reluctance to prioritize or label terms and concepts, and the focus on community-based, local needs, it is comprehensible why we do not see a push towards our model. However, if EcoHealth is to attain its goal of improving the health and well-being of humans, animals, and the environment from local to global scales, it would be beneficial for the field of EcoHealth to conceptually engage with the different types of determinants and their indicators, as well as other key areas such as health inequity and global health governance.

### 3.4. Limitations

We use the term “limitations” to indicate a boundary around the focus of our study rather than a weakness of the study. A boundary is that our sample size is relatively small and homogeneous in that we sought participants from a Canadian context, that were academics within the EcoHealth community. However, a small sample size allows for in-depth engagement and analysis, while a homogeneous sample fosters ‘information-rich’ accounts [22].

### 3.5. Future Directions

In our sample, there was one participant who was both an EcoHealth academic as well as a health practitioner and we found her views to be quite unique. Future research could explore the views of health practitioners to examine the ways in which they engage with the determinants of health. The structure of our assessment also drew attention to the impacts of colonialism on Indigenous people. In future work, we could consider the impacts of colonialism on descendants of the colonists as well. Finally, given our participant accounts, future research might be to engage with universities on how they teach EcoHealth in relation to the determinants of health across national contexts.

## 4. Conclusions

EcoHealth’s ability to improve human, animal, and environmental health and well-being is influenced by its ability to identify and integrate the different determinants of health to address human, animal and environmental health and well-being [2]. Our findings support our previously identified gap of engagement with determinants of health [21], and indicate a reluctance to prioritize different determinants of health, and to label terms and concepts, as well as a focus on community-based, local needs. As we consider a future with rapid and unsustainable changes [10], the explicit identification and integration of each of the different types of determinants of health within EcoHealth and their application to humans, animals and the environment is imperative for the EcoHealth community to achieve its goals and to act as an exemplar for others that engage with complex health problems.

## Figures and Tables

**Table 1:** Participant Characteristics of Canadian EcoHealth Community Members.

**Gender**	**Male**	2
	**Female**	5
**Age**	**25–34 Years**	4
	**35–44 Years**	1
	**45+ Years**	2
**Residence**	**Western Canada**	4
	**Eastern Canada**	3
**Educational Background**		Community health
		Ecological health
		Environmental studies
		Health policy management & evaluation
		Health sciences
		Human ecology
		International development
		Medicine
		Nursing
		Political science
		Public health
		Recreation Therapy
		Sciences
		Social Behavioral Sciences
		Sociology
**General Occupation**		Adjunct professor
		Adult teaching and learning
		Associate professor
		Grad student
		Health researcher
		Post-doctoral fellow
		Research assistant

**Table 2:** Overview of the Themes and Subthemes that emerged from the views of Canadian EcoHealth Community Members.

Themes	Subthemes		Sub-Subthemes
1. Recognition of Definitional Issues—The Determinants of Health	1.1	The Social Determinants of Health	
	1.2	The Ecological and Environmental Determinants of Health	1.2.1 The Ecological Determinants of Health
			1.2.2 The Environmental Determinants of Health
			1.2.3 The Ecological/Environmental Determinants of Health
	1.3	The Political Determinants of Health	
	1.4	The Cultural Determinants of Health	
	1.5	Recognition of Definitional Issues—A Synopsis	
2. The System	2.1	Ecosystem	
	2.2	Integrating Individual Elements	2.2.1 Systems Thinking and Understanding
3. Multiple Realities	3.1	‘It Depends’	
	3.2	Normative	
	3.3	Labelling	
4. Power	4.1	Blind to Political Power	
	4.2	Colonialism	
5. Transdisciplinary	5.1	EcoHealth and Transdisciplinarity	5.1.1 Public Health
	5.2	Different Knowledges	5.2.1 Traditional Ecological Knowledge

## Appendix A. Interview Guide

*Demographics:*

1. What is your name?
2. What is your gender?
3. What is your age?
4. Where do you reside?
5. What is your education? Preferably every degree.
6. What is your affiliation or occupation?
  - How might it relate to EcoHealth?

*EcoHealth:*

1. What do you think EcoHealth is?
2. What is EcoHealth’s purpose?
3. In your opinion, what is one major achievement of EcoHealth and what might be some challenges or risks to EcoHealth?
4. What is your vision of the future of EcoHealth?

*Determinants of health:*

1. Briefly, how would you define health and well-being?
2. Briefly, how would you define determinants of health?
3. What do you think the social determinants of health are as a concept?
  - In what ways do you think EcoHealth does or should engage with the social determinants of health?
  - What determinants would you list under the social determinants of health?
  - Which specific social determinants do you think EcoHealth does or should engage with?
  - Do you engage with the social determinants of health in your work? If so, with which social determinants and why?
4. Do you think the ecological and environmental determinants of health differ from each other?
  **Depending on the answer to Question 4, I will ask Questions 5 and 6 or have them combined**
5. What do you think the ecological determinants of health are as a concept?
  - In what ways do you think EcoHealth does or should engage with the ecological determinants of health?
  - What determinants would you list under the ecological determinants of health?
  - Which specific ecological determinants do you think EcoHealth does or should engage with?
  - Do you engage with the ecological determinants of health in your work? If so, with which ecological determinants and why?
6. What do you think the environmental determinants of health are as a concept?
  - In what ways do you think EcoHealth does or should engage with the environmental determinants of health?
  - What determinants would you list under the environmental determinants of health?
  - Which specific environmental determinants do you think EcoHealth does or should engage with?
  - Do you engage with the environmental determinants of health in your work? If so, with which environmental determinants and why?
7. What do you think the political determinants of health are as a concept?
  - In what ways do you think EcoHealth does or should engage with the political determinants of health?
  - What determinants would you list under the political determinants of health?
  - Which specific political determinants do you think EcoHealth does or should engage with?
  - Do you engage with the political determinants of health in your work? If so, with which political determinants and why?
8. What do you think the cultural determinants of health are as a concept?
  - In what ways do you think EcoHealth does or should engage with the cultural determinants of health?
  - What determinants would you list under the cultural determinants of health?
  - Which specific cultural determinants do you think EcoHealth does or should engage with?
  - Do you engage with the cultural determinants of health in your work? If so, with which cultural determinants and why?
9. How would you rank the 4 (or 5) different types of determinants of health covered in this survey in relation to their importance for EcoHealth?
10. Now that we have talked about several types of determinants, how do they or should they work together within EcoHealth?
